# Drug repurposing and prediction of multiple interaction types via graph embedding

**DOI:** 10.1186/s12859-023-05317-w

**Published:** 2023-05-17

**Authors:** E. Amiri Souri, A. Chenoweth, S. N. Karagiannis, S. Tsoka

**Affiliations:** 1 Department of Informatics, Faculty of Natural, Mathematical and Engineering Sciences, King’s College London, Bush House, London, WC2B 4BG UK; 2 St. John’s Institute of Dermatology, School of Basic and Medical Biosciences, Guy’s Hospital, King’s College London, London, SE1 9RT UK; 3 Breast Cancer Now Research Unit, School of Cancer and Pharmaceutical Sciences, Guy’s Cancer Centre, King’s College London, London, SE1 9RT UK

**Keywords:** Drug-target interaction, Drug discovery, Drug repurposing, Network embedding, Machine learning

## Abstract

**Background:**

Finding drugs that can interact with a specific target to induce a desired therapeutic outcome is key deliverable in drug discovery for targeted treatment. Therefore, both identifying new drug–target links, as well as delineating the type of drug interaction, are important in drug repurposing studies.

**Results:**

A computational drug repurposing approach was proposed to predict novel drug–target interactions (DTIs), as well as to predict the type of interaction induced. The methodology is based on mining a heterogeneous graph that integrates drug–drug and protein–protein similarity networks, together with verified drug-disease and protein-disease associations. In order to extract appropriate features, the three-layer heterogeneous graph was mapped to low dimensional vectors using node embedding principles. The DTI prediction problem was formulated as a multi-label, multi-class classification task, aiming to determine drug modes of action. DTIs were defined by concatenating pairs of drug and target vectors extracted from graph embedding, which were used as input to classification via gradient boosted trees, where a model is trained to predict the type of interaction. After validating the prediction ability of DT2Vec+, a comprehensive analysis of all unknown DTIs was conducted to predict the degree and type of interaction. Finally, the model was applied to propose potential approved drugs to target cancer-specific biomarkers.

**Conclusion:**

DT2Vec+ showed promising results in predicting type of DTI, which was achieved via integrating and mapping triplet drug–target–disease association graphs into low-dimensional dense vectors. To our knowledge, this is the first approach that addresses prediction between drugs and targets across six interaction types.

**Supplementary Information:**

The online version contains supplementary material available at 10.1186/s12859-023-05317-w.

## Introduction

Drug discovery remains a time-consuming and costly process, with low success rate [1, 2]. Early studies on drug effects through trial-and-error procedures have been superseded by advances in chemical genomic research allowing complex analyses and insight in mechanisms of drug actions [3]. Due to the intrinsic complexity of molecular structures, the vast majority of drugs tend to interact with multiple targets either in a unique disease pathway or across multiple pathways [4], referred to as polypharmacology [5]. This observation has changed the drug design philosophy of “one gene, one drug, one disease” paradigm and fuelled consecutive work in drug discovery [5]. Reaping the effects of polypharmacological approaches to discover unknown off-targets for approved drugs and allow existing drugs to be applied for the treatment of another indication, drug repurposing has emerged as a promising route to drug discovery [6].

Drug repurposing can offer a shorter path in drug discovery, bypassing several steps in drug development [2, 7], and can improve understanding of drug mechanisms of action and drug side effects [8]. Drug repurposing strategies have shown successful applications in the past [1], with potential advantages and approved examples outlined in relevant reviews (for example see papers by Pushpakom et al. [9] and Ashburn et al. [10]). The first main step is finding possible drug–target interactions (DTIs) outside the original scope of a drug [9], which it is a crucial task in drug discovery. In addition to predicting the presence of DTIs, it is also important to determine the type of interaction, as different types of interactions can have varying therapeutic outcomes by increasing or decreasing expression and reaction [11]. Drug development relies heavily upon targeting “druggable” genes, which include more than 4500 genes in the human genome [12, 13]. However, only a small number of these genes are targeted by approved drugs and many interactions between genomic and chemical spaces remain unknown [14]. Although some DTIs have been identified via serendipitous or rational observations, they are not efficient in practice due to the vast search space that needs to be covered in lab experimentation [15]. These limitations strengthen the case for computational investigation of DTIs, as means of narrowing the search space and proposing the most promising cases on which to focus drug development.

Moreover, in pathological processes of complex diseases, multiple genes and pathways may be implicated [16, 17]. Targeting druggable genes (known as targeted therapy) is a key strategy in treating complex diseases, including cancer [18]. Recently, research on drug repurposing has shown that disease-specific gene biomarkers, as well as associations between drugs and diseases, can be used to accurately predict drugs for new diseases through identifying novel off-target interactions [11, 19–21]. However, most of the existing studies focused on either drug-disease or drug–target interactions as two isolated tasks and did not take account of the relationships between these [18]. Therefore, systematic integration of relationships represented in gene, disease, and drug networks can lead to more reliable new DTI prediction [2, 22]. Additionally, these developments can significantly reduce and refine experimental laboratory costs and processes, as well as the risk of failure in drug development [11].

In terms of methodological avenues to predicting DTIs, traditionally molecular docking and ligand-based methods have been used [23]. However, docking-based methods rely on the availability of protein 3D structural information, which can be challenging for large-scale prediction [7, 22], while ligand-based methods are not accurate in cases where only a small number of known binding ligands are available [24]. Recently, machine learning-based (ML) methods have attracted much interest in drug repurposing, due to their ability to analyze large numbers of DTIs efficiently by extracting latent association patterns [19, 25]. A wide variety of ML methods in drug repurposing have been proposed and summarized previously [26–28].

Similarity-based methods rely on the key underlying assumption that similar drugs developed for similar diseases tend to target similar proteins and constitute the most widely applied strategy to integrate biological networks [7, 29]. These methods can integrate different large-scale associations of genes, diseases, and drugs into a heterogeneous graph [30, 31], and then formulate DTI prediction as a link prediction problem in graph analysis [14, 18]. The “2vec” (short for “to vector”) methods form an important category of embedding methods (e.g. “graph2vec”, “node2vec” etc.) and have shown promising potential in representing input features in ML tasks by mapping graph structural properties to low dimensional vectors [32]. Recently, different embedding methods have been proposed for predicting new DTIs [3, 18, 25, 32, 33]. Although these methods achieved promising results, they can only predict binary interactions between drugs and target proteins and cannot identify the type of interaction (i.e. *action* type such as activation, expression, reaction, etc.) or the effect of a drug on its target (i.e. *degree* type such as increase/decrease in expression, etc.), which would be essential in the process of targeted treatment and in understanding drug action for drug repurposing [11, 21].

In targeted therapy for cancer [34], the current therapeutic debacle is discovering and targeting cancer biomarkers. Carcinogenesis is the result of mutations in oncogenes and/or tumour suppressor genes [35]. Molecular targeted therapies that inhibit oncogenes and/or activate tumour suppressor gene products can limit or stop tumour progression [36]. Despite efforts in pharmaceutical research to develop new drugs to target these genes, the implementation of discovered drugs in clinical practice has lagged far behind expectations. Therefore, drug repurposing should be considered as a promising strategy to address the unmet need for efficacious cancer therapies. One of the main advantages of this approach is the availability of toxicity, pharmacodynamic, pharmacokinetic profiles for these drugs have already been established [37].

In this work, we propose an ML-based computational pipeline for drug repurposing, DT2Vec+, that integrates the triplet associations of drug–target–disease data to a heterogeneous graph by incorporating drug–drug and protein–protein similarity networks, together with verified drug-disease and protein-disease associations [19, 38]. To extract features from the network, the three-layer heterogeneous graph was mapped to low dimensional vectors using node2vec. The DTI prediction problem was formulated as a multi-label, multi-class classification to determine drug modes of action, defined as “increases^expression”,”decreases^expression”,”decreases^reaction”,”increases^reaction”,”increases^activity”,”decreases^activity”. In previous work, Wang and Zeng [11] proposed an ML-based model using Boltzmann machines to predict three types of DTIs (binding, activation and inhibition), so to our knowledge DTVec+ is the first method investigating six drug–target interaction degrees and types. DTIs were defined by concatenating pairs of drug and target vectors extracted from graph embedding, which were used as input to gradient boosted trees (XGBoost) to train a model for interaction type prediction. Cross-validation was used to evaluate performance and a comprehensive analysis of unknown DTIs was conducted to evaluate results in terms of the degree and type of interaction. Our results have also been appraised in terms of case studies for potential drugs that target important oncogenes and their medical potential is discussed.

## Materials and methods

**Fig. 1:**
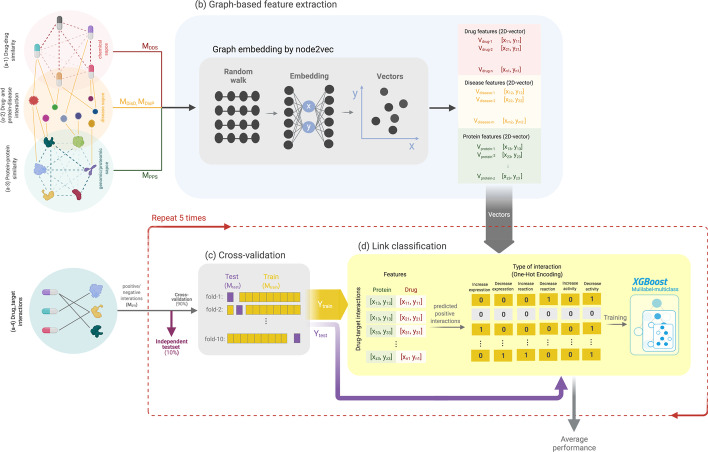
DT2Vec+ pipeline. (**a**-1,2,3) integrating drug–drug (DDS) and protein–protein (PPS) similarity graphs with drug-disease (DDis) and disease-protein (DisP) association graph as input of embedding method to low dimensional vectors. (**a**-4) Drug–target interaction graph with different edge types. **b** Graph-embedding developed by node2vec to map nodes to vectors (in this figure, nodes are shown mapped to 2D-vector, x and y). **c** Known drug–target interactions (six types of interactions) were divided into 10% independent dataset (external testset) and 90% internal test and train (tenfold cross-validation). **d** Drug and protein vectors were concatenated and labelled using one-hot encoding and an XGBoost model was trained on each label using cross-validation. The best model over the tenfold cross-validation on the internal testset was selected and applied on the external testset. The XGBoost model in c, d was repeated 5 times and the average performance of internal and external testsets was reported

Figure 1 presents an overview of the computational framework in DT2Vec+, which includes network integration, feature extraction, implementation of the proposed methodology and evaluation using cross-validation. Categorical labels for degree and type of interaction were converted to binary vectors through one-hot encoding, and one-vs-rest strategies were used to train the model against each label. The average performance of all models was measured on external test-sets.

### Dataset and similarity networks

Drug–target interactions (M_DTI_, Fig. 1: a-4), disease-protein (M_DisP_), and disease-drug (M_DisD_) associations (Fig. 1: a-2) were extracted from the Comparative Toxicogenomics Database (CTD) [38]. Since only drug MeSHid was provided in the Comparative Toxicogenomics Database (CTD), we used DrugBank [39] and ChEMBL [40] datasets to find drug SMILES and phase which shows molecule connectivity and chirality, and trials testing stage of drug respectively. Additional file 1: Fig. S1 shows steps performed to collect the dataset. Only approved phase-4 drugs with therapeutic evidence were selected for further repurposing analysis. Table 1 shows details of the selected dataset.

**Table 1:** Dataset details

Dataset	Frequency
Protein	1141
Drug	280
Disease	589
Disease-protein interaction	1305
Disease-drug interaction	301
Drug–target (protein) interaction	4086
Drug–target pairs	3502

The CTD dataset categorised DTIs into different degrees and types of interaction based on published references as positive interactions. Selecting experimentally validated negative DTIs is an important point in developing an accurate model for DTI prediction. Therefore, the ChEMBL dataset was used to select 348 validated negative DTIs labelled as *‘inactive’* [41]. The known DTIs named ‘*development dataset’* which is used for developing the ML model, comprise 4086 interactions between 3502 drug–target pairs assigned to six categories as labels, namely “increases^expression” (1392),”decreases^expression” (708),”decreases^reaction” (158),”increases^reaction” (1017),”increases^activity” (396), and”decreases^activity” (415). DTIs have one (​​2922), two (576), or three (4) types of interactions. Importantly, cases with more than one interaction type were ‘multi-label’ and among these DTIs that have two interaction degrees with the same action type (e.g. increases^expression and decreases^expression) were considered as ambiguous, and removed from further analysis. All possible drug–target pairs without known interactions were defined as an *‘experimental dataset’* and used for drug repurposing analysis.

To calculate protein–protein similarity (PPS), M_PPS_ (Fig. 1: a-3) by sequence alignment [42], the sequences of target proteins were extracted from UniProt [43]. The parallelised version of protein similarity calculation was implemented using the “protr” package in R 3.3 [44]. Drug similarity measures were defined based on 166 structural fingerprints from canonical SMILES using MACCS [45]. Then, the drug–drug similarity (DDS) network, M_DDS_ (Fig. 1: a-1) was calculated based on the Tanimoto coefficient in the range of 0 to 1 [46] which was implemented using Open Babel [47] in Python 3.7.3 [48]. A triplet drug–target–disease association graph was generated by integrating four networks of M_DisP_, M_DisD_, M_PPS_, and M_DDS_ by matching similar proteins and drugs on M_PPS_ and M_DisP_ and M_DisP_ and M_DDS_ respectively (Fig. 1a). The new graph consists of 692,177 edges between 2011 nodes (1141 proteins, 280 approved drugs and 589 diseases).

### Network-based feature extraction using node2vec

Changing the format or shape of raw data to extract informative and discriminative features, known as feature extraction, is an important step for an effective ML model. Graph embedding methods can create powerful representation structural information by converting the topological properties of a heterogeneous network to a set of features in low-dimensional space [25, 49] that can be used as input to a predictive model. In this work, we used node2vec [50], a neural network-based node embedding method to automatically map nodes in the drug–target–disease association graph into a 100-dimensional vector which was reported as the best threshold to accurately preserve graph information [25, 49–51]. Recent machine learning research has shown that node2vec is a superior method for node embedding compared to other existing state-of-the-art methods [51, 52]. In a recent study, node2vec demonstrated encouraging outcomes in predicting drug–target interactions (DTIs) by converting drug, protein, disease, lncRNA and miRNA association networks into vectors [49, 50]. Figure 1b illustrates the embedding process, where drug (V_drug_), protein (V_protein_) and diseases (V_disease_) nodes are mapped to two-dimensional vectors, as an example. Node2vec was implemented in python 2 using publicly available GitHub source code (https://github.com/aditya-grover/node2vec) [50].

### Data-splitting, cross-validation, and performance evaluation metrics

Various validation methods may be used for drug–target interaction prediction models [53]. Among these, cross-Validation (CV) methods are preferred due to their robustness in estimating how a model generalizes. Validation was conducted on internal and external testing as follows. All known DTIs (M_DTI_) in the ‘*development dataset’* were split into 80% training and 10% validation set to train and select the best model. The external testset (i.e. the remaining 10% of the data) was used to evaluate the performance of the model and was blind to the process of developing the model. We applied a tenfold CV (Fig. 1c) which involved randomly splitting the data into 10 partitions and iteratively selecting each partition as the testing data and training the model on the remaining partitions and this procedure was repeated five times.

Choosing the right metrics for evaluating the performance of the model is important, but can be challenging depending on the underlying assumptions. The aim of the DTI prediction model is to report positive interactions among all unknown drug–target pairs, so that highly positive interactions can be validated experimentally, therefore a low false-positive rate is desirable. In this case, the Precision metric (true positive/(true positive + false positive)) can better reflect performance. However, in general a DTI prediction model should have high precision without sacrificing other metrics. Therefore, to evaluate the performance of the model, average Precision, f1-score, and accuracy across all cross-validation sets on external test sets were calculated.

### Multi-label, multi-class ML-based link classification

DTIs were represented as a 200-dimensional vector based on a strategy of concatenating the drug and target embedding features [32] and labelled based on six types of interaction, in terms of affecting expression, reaction rate or binding affinity (increases^expression, decreases^expression, decreases^reaction, increases^reaction, increases^activity, and decreases^activity). The DTI prediction problem was formulated as a multi-label, multi-class link classification built on XGBoost [54]. XGBoost is a stochastic gradient boosting algorithm which combines weak ensemble decision trees and was selected due to its high speed, accuracy, and ability to handle imbalanced datasets [12]. Moreover, by taking advantage of XGBoost in returning the prediction probability score, we were able to rank DTIs based on the confidence of the model. We used one-hot encoding to create six binary vector labels and different models were trained based on one-vs-rest strategies for each label type (Fig. 1d). Training data was balanced by over-sampling via Synthetic Minority Over-sampling Technique (SMOTE) [55]. Oversampling is a technique used to balance sample numbers in imbalanced datasets by generating synthetic data for the minority class, and the number of samples increased in each class depends on the specific implementation and the degree of imbalance [55]. Grid-search was performed on training-set samples within each cross-validation fold to find the best set of hyperparameters. The model was implemented in Python 3.7.3, using XGBoost 0.90 with hyperparameters of maximum tree depth = r, subsample ratio = 1, gamma = 1, minimum child weight = 2, early stopping = 20 and learning-gamma rate = 0.01.

### Novel DTIs extraction and drug repurposing

After validating the performance of the proposed method using cross-validation, in order to detect novel DTIs a two-step prediction was applied to unknown interactions (named as ‘experimental dataset’). First, experimentally validated negative interactions and DT2Vec [56] were used to predict highly positive interactions with a probability score >  = 0.95%. DT2Vec is a machine learning pipeline that formulates the problem of deriving new drug–target interactions as binary (positive or negative) link prediction [56]. Then DT2Vec+ was applied on positive interactions to identify the six types of DTIs based on the triple association graph. A small number of the novel DTIs was analysed for target treatment. In this work, we also focus on several cancer gene biomarkers and establish a connection between our repurposing prediction results and potential role of the predicted DTI in cancer drug development.

## Results

**Fig. 2:**
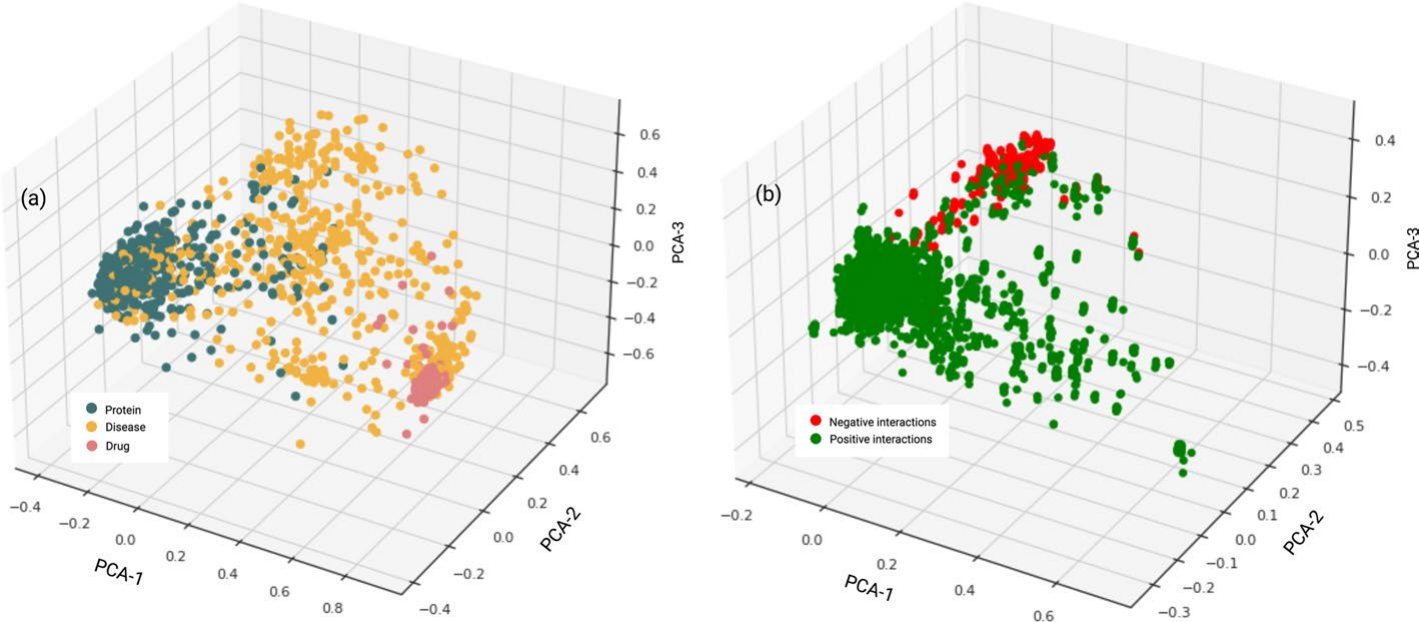
Drugs, diseases, proteins and DTI visualisation. **a** PCA of drugs, diseases, and proteins vectors extracted from heterogeneous association graph. **b** PCA of DTIs mapped vectors by concatenating drug–target vectors

DT2Vec+ was developed based on concatenating drug–target pair vectors extracted from a heterogeneous association graph consisting of three node types of drug, protein and diseases, connected through four edge types (DDS, PPS, DDis, and DisP associations) as shown in Fig. 1a,b. Figure 2a shows Principal Component Analysis of mapped vectors associated with the three node types. DTIs were defined by concatenating each drug–target pair vector and labelling based on the type of interaction. Six models were trained for each type of DTI based on one-vs-rest strategy. Performance was assessed via tenfold cross-validation repeated five times as described previously, and average results for each label are shown in Table 2. When applied to external test sets, the proposed method achieved average Accuracy, f1-score, and Precision of 77.09% (0.02), 74.39% (0.02), and 84.58% (0.01), respectively.

**Table 2:** DT2Vec+ performance metrics (variance shown in parentheses)

	Mean accuracy	Mean f1_score	Mean precision
increases^expression	73.35% (0.01)	73.34% (0.02)	73.27% (0.01)
decreases^expression	74.68% (0.01)	72.08% (0.02)	80.23% (0.01)
increases^reaction	77.2% (0.03)	71.84% (0.03)	91.78% (0.02)
decreases^reaction	77.8% (0.02)	76.68% (0.02)	80.79% (0.02)
increases^activity	75.63% (0.01)	70.26% (0.03)	89.95% (0.01)
decreases^activity	83.88% (0.02)	82.17% (0.03)	91.46% (0.01)
Total	77.09% (0.02)	74.39% (0.02)	84.58% (0.01)
Binary (active/inactive)	95.81% (0.02)	96% (0.02)	92.54% (0.03)

After validating the performance of DT2Vec+, in order to extract new interaction types in unknown DTIs (‘experimental dataset’), we performed a two-step prediction of binary (activation or inactive) and multi-class, multi-label (interaction type) classification. Figure 2b shows the PCA of positive and negative DTIs defined based on embedded vectors. In the first step, a model was trained to stratify positive and negative DTIs. The performance of the model on the external-test set is summarised in Table 2. This achieved higher than 90% on all metrics. Then, the model was applied to unknown DTIs to find positive interactions. Interactions with positive probability score of 95% or higher were selected as highly positive interactions which then were used as input of DTI2Vec+ to identify the type of interaction.

**Fig. 3:**
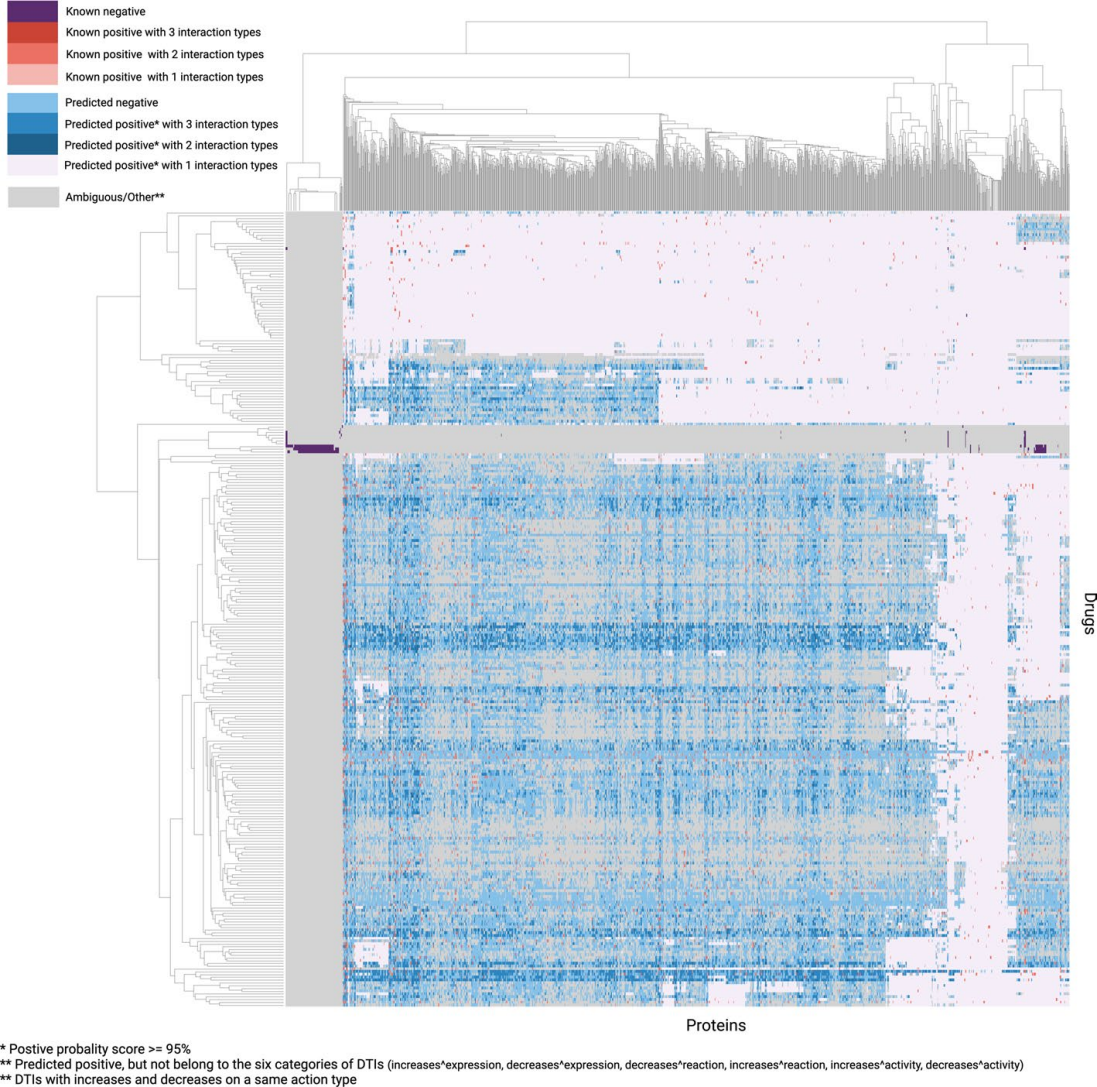
Interaction type of all drug–target pairs. The heatmap shows the mapping of known DTI interactions (red and purple) and predicted interactions (blue and lilac). Each interaction can have multiple types of interactions, which were coloured darker

**Fig. 4:**
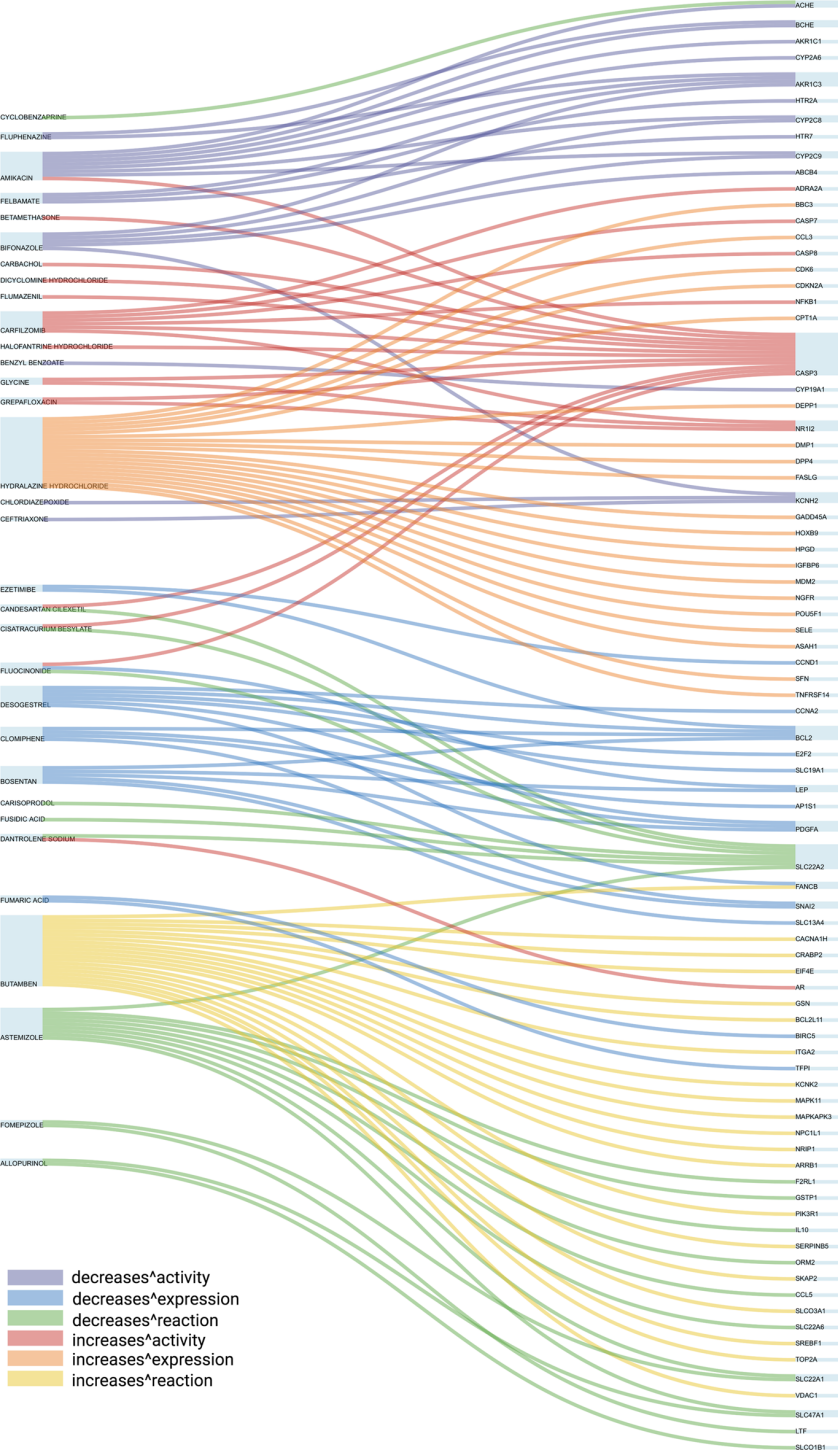
Top novel drug–target interactions. Top 20 DTIs predicted using DT2Vec++ for each type of interaction coloured based on the type of interaction

Figure 3 summarises labels of all drug–target pairs which take the form of either *known* (coloured red for positive and purple for negative) or *predicted* (coloured blue for positive and lilac for negative) interactions. DTIs with multiple labels were marked darker compared with the interactions with one label type. We predicted 18,736 and 787 DTIs with two and three label types, respectively. The top 20 new predicted DTIs for each interaction type are shown in Fig. 4. However, there were some highly positive interactions that did not belong to any of our six labels or which had more than three labels (ambiguous/other interactions), which we excluded from further analysis. For example, Amikacin, Desogestrel, and Astemizole may interact with multiple proteins and decrease the activity, expression, and reaction respectively while Carfilzomib, Hydralazine hydrochloride, and Butamben may increase the activity, expression, and reaction of multiple proteins. The novel high-scoring DTIs proposed by this method can narrow down the search space in a wet-laboratory experiment towards finding drugs able to target a specific protein.

## Discussion

Drug repurposing holds promising potential in reducing failure risks and costs of developing new drugs. Finding appropriate drugs that can interact with a specific target is a pivotal step in drug repurposing strategies. However, due to the massive number of potential interactions, it is almost impossible to base drug discovery solely on wet-lab experiments without the help of computational methods and virtual screening which are able to reduce the number of potential interactions for downstream experimental validation. In this work, we report an ML model, DT2Vec+, to predict the type of drug–target interaction based on features extracted from a heterogeneous drug-disease-target graph using an embedding approach. The problem of drug–target link prediction was formulated as multi-label, multi-class classification and the method was able to stratify DTIs into six different interaction types, with performance higher than 75% on average in test sets across validation metrics. Our pipeline was used as a tool for the identification of targeted treatments by selecting potentially targetable oncogenes and predicting some drug candidates that affect the activity of proteins associated with breast and other cancers, as discussed next.

BIRC5 (Survivin) has been reported to be an important biomarker for breast cancer [17, 57], with high expression of BIRC5 correlated with worse survival, and is a promising target for drug discovery and breast cancer therapeutics. BIRC5 encoding survivin is involved in carcinogenesis by influencing cell division and proliferation and inhibiting apoptosis [58]. Therefore, downregulation of BIRC5 can act as an inhibitor of tumour cell migration and invasion through the PI3K/Akt signaling pathway. In the CTD dataset, there are seven known DTIs where the drugs can decrease expression of BIRC5, namely Digitoxin (CHEMBL254219), Phenylbutanoic acid (CHEMBL1469), Bexarotene (CHEMBL1023), Ciclopirox (CHEMBL1413), Danthron (CHEMBL53418), Etodolac (CHEMBL622), Ethacrynic acid (CHEMBL456). Based on DT2Vec+ prediction results, Aminolevulinic acid (CHEMBL601), Cladribine (CHEMBL1619), Fumaric acid (CHEMBL503160), and Bosentan (CHEMBL957) were among the top drugs predicted to decrease the expression of BIRC5. Aminolevulinic acid and Cladribine have been investigated for cancer treatment in several clinical trials. Recently, a study investigating the relationship between survivin expression and melanoma after using Aminolevulinic acid treatment showed inhibition of melanoma growth in mice by downregulating survivin expression, which prolonged the survival of melanoma-bearing mice [59]. Aminolevulinic acid photodynamic therapy also induced apoptosis in cervical cancer cells in *vitro* and in *vivo* by decreasing survivin expression [60]. Cladribine also inhibited cell proliferation and induced apoptosis in multiple myeloma cells in *vitro* [61]. Cladribine also inhibited cell proliferation and induces apoptosis in multiple myeloma cells in *vitro* [62, 63]. Cladribine has been shown to increase progression-free survival and median time to second treatment in chronic lymphocytic leukaemia patients [61]. Finally, Bosentan has been reported to inhibit breast carcinoma metastasis to bone tissue in a skinfold chamber model [64].

BCL-2 is a key protein regulator of apoptosis and is overexpressed in many cancer types [65]. BCL-2 has been reported to be frequently expressed in breast cancer [66] and can reduce the effectiveness of chemotherapy [67]. There is an inverse correlation between the expression of BCL-2 and mutated p53, an important tumour suppressor gene, which in turn leads to increased cell proliferation rates and poor outcomes [66]. Targeted therapy against BCL-2 may improve the effects of chemotherapy in breast cancer patients [66]. Clioquinol (CHEMBL497), Adenosine (CHEMBL477), Diacerein (CHEMBL41286), Azelaic Acid (CHEMBL1238), Dequalinium (CHEMBL333826), Azelastine (CHEMBL639) and Bazedoxifene (CHEMBL46740) are DT2Vec+ predicted drugs that might be able to decrease the expression of BCL-2. Research showed that Clioquinol reduced the viability of eight different human cancer cell lines by inducing cell death through apoptotic pathways [68]. It also induced autophagy in leukaemia and multiple myeloma cell lines. Downregulated expression of BCL-2 can inhibit the interaction between Beclin 1 and BCL-2 and stimulate autophagy [69]. Adenosine induces cell cycle arrest and apoptosis in ovarian cancer cell lines by down-regulating BCL-2 [70]. Adenosine also induced apoptosis in different cancer types such as breast [71], leukaemia [72], gastric [73], colon [74], melanoma [75], and head and neck cancer cell lines, and suppressed BCL-2 expression [75].

Diacerein is another approved drug that has been reported to exert anti-proliferative effects on breast cancer cell lines, induced apoptosis and decreased the expression of BCL-2 [76]. Azelaic acid has cytotoxic action on many tumour cells and antileukemic activity in different types of acute myeloid leukaemia cells. It increases Notch expression which leads to the loss of BCL-2 expression [77]. Recently, Dequalinium showed promising results in *vitro* and in *vivo*, inhibiting the growth and proliferation of human glioma cells by decreasing BCL-2 expression [78]. Dequalinium injection into tumour-bearing mice inhibited the growth of human colon cancer cells [79]. The drug was able to prolong the survival of mice with bladder carcinoma cells [80], and inhibit the growth, migration and invasion of melanoma cells in *vitro* [81]. Dequalinium also reduced acute myeloid leukaemia cell activity, proliferation, induced apoptosis and increased survival of rats with ovarian cancer [82]. In *vitro* and in *vivo* analyses showed that Azelastine could decrease levels of BCL-2 and inhibit colorectal cancer cell proliferation [83]. Bazedoxifene has been used in clinical trials for treating pancreatic and breast cancer. Bazedoxifene is now being repositioned as a new strategy for treating multiple cancer types (such as breast cancer, pancreatic cancer, colon cancer, etc.) by downregulating anti-apoptotic proteins such as BCL-2 [84].

MYC is involved broadly in many cancer types and its expression was estimated to be deregulated in up to 70% of human cancers [85]. High levels of MYC were linked to aggressive prostate cancer and triple-negative breast cancer. Two FDA approved drugs, Dihydroergotamine (CHEMBL1732) and Indinavir Sulfate (CHEMBL1735), which are predicted by the DT2Vec+, might be able to target MYC and decrease its expression. In a mouse xenograph model, Dihydroergotamine could suppress the growth of MYC-dependent human acute myeloid leukaemia, and in this study, MYC was the most statistically repressed gene by Dihydroergotamine [86]. Indinavir blocked tumour formation in an angiogenic tumour model, and this inhibition was associated with inhibition of cell invasion but not cell proliferation or cell survival. Indinavir sulfate was also effective at inhibiting the growth of various human tumour xenografts, including lung, breast, hepatocarcinoma and colon adenocarcinoma, and human tumours of haematopoietic cell origin. The drug effectively blocked the invasion of a basement membrane by lung, breast, colon adenocarcinoma [87].

Finally, STAT3 is constitutively activated in more than 40% of breast cancers and is thought to promote breast tumour progression [88]. Therefore, drugs that can reduce the activity of STAT3 have been attracting more attention. Using the DT2Vec+ methodology, four drugs were proposed to target STAT3, namely, Amsacrine (CHEMBL43), Phenylbutanoic Acid (CHEMBL1469), Doxazosin (CHEMBL707) and Capecitabine (CHEMBL1773). Amsacrine is an anti-cancer drug that showed significant activity against human acute leukaemia and it is currently approved for treatment [7]. Phenylbutanoic Acid has been reported to be able to inhibit cell proliferation by inducing apoptosis, cell cycle arrest, and senescence in colon, gastric and breast cancers. However, clinical trials with Phenylbutanoic acid in solid tumours showed no obvious benefit [89]. Doxazosin suppressed the growth of ovarian carcinoma cells and additively enhanced apoptotic cell death by IFN treatment and its effects were potentiated by reducing phosphorylation of STAT3 [90]. Doxazosin also could significantly inhibit prostate and bladder cancer cell growth in *vitro* [91, 92]. Capecitabine is a currently approved chemotherapy drug, and a meta-analysis of clinical trials of patients with triple-negative breast cancer treated with capecitabine in combination with neoadjuvant or adjuvant chemotherapy demonstrated improved survival [93]. Studies in triple-negative breast cancer patient-derived xenograft (PDX) models also have shown Capecitabine as an efficient chemotherapy agent [94].

## Conclusion

In silico prediction of DTIs is an efficient approach for drug repurposing. Various methods have been proposed to predict the interactions between drugs and targets in a binary format (active or inactive), but determining drug mode of action has remained elusive. Importantly, deciphering the type of interaction in targeted treatment is an important step in developing an effective method. In this work, we reported DT2Vec+, which—to our knowledge—is the first ML-based framework to predict six types of DTIs, by integrating associations between drugs, diseases, and proteins into a heterogeneous graph consisting of DDS, PPS, DDis, and DisP edges. The triple association graph was mapped to low dimensional vectors using graph embedding, and DTIs were defined based on concatenating drug–target vectors. We show that this pipeline achieved high performance on external test sets and was applied to unknown DTIs to predict the type of interaction. DT2Vec+ can offer a means to improve and support precision targeted treatments by selecting the drug candidates that can bind to specific targets in desired action modes. Predicting potential drugs provides an alternative approach to narrow down the search space that can be investigated in follow-up laboratory experiments. This approach can significantly reduce wet-laboratory work and experimental cost, but most importantly it can refine downstream experimental validation.

We note that the benefits conferred by our methodology stem from its capability to incorporate heterogeneous chemical and genomic data into a unified space, in addition to the fact that machine learning algorithms can handle numerical input features well. Predictions generated by our method can be used for virtual screening of novel DTIs at large scale. Although we have obtained promising results in predicting different types of DTIs using the drug–target–disease association network, this first report provides the foundations for the model reported here to be expanded by integrating more biological information into the association network.

## Supplementary Information

**Additional file 1. Fig. S1**: steps performed to collect the dataset.

## Data Availability

The datasets analysed during the current study are available in the ChEMBL repository (https://www.ebi.ac.uk/chembl/), CTD dataset (http://ctdbase.org/). The code is available at https://github.com/elmira-amiri/DT2VecPlus.

## Abbreviations

CV: Cross-validation
DDS: Drug–drug similarity
DTI: Drug target interaction
ML: Machine learning
PCA: Principal component analysis
PPS: Protein–protein similarity
PDX: Patient-derived xenograft
Ddis: Drug-disease
DisP: Disease-protein in the future
