# Supplementary figures and images for: Drug repurposing and prediction of multiple interaction types via graph embedding
Source: BMC Bioinformatics. 2023 May 17;24:202. doi: 10.1186/s12859-023-05317-w (PMC10190044; doi:10.1186/s12859-023-05317-w)

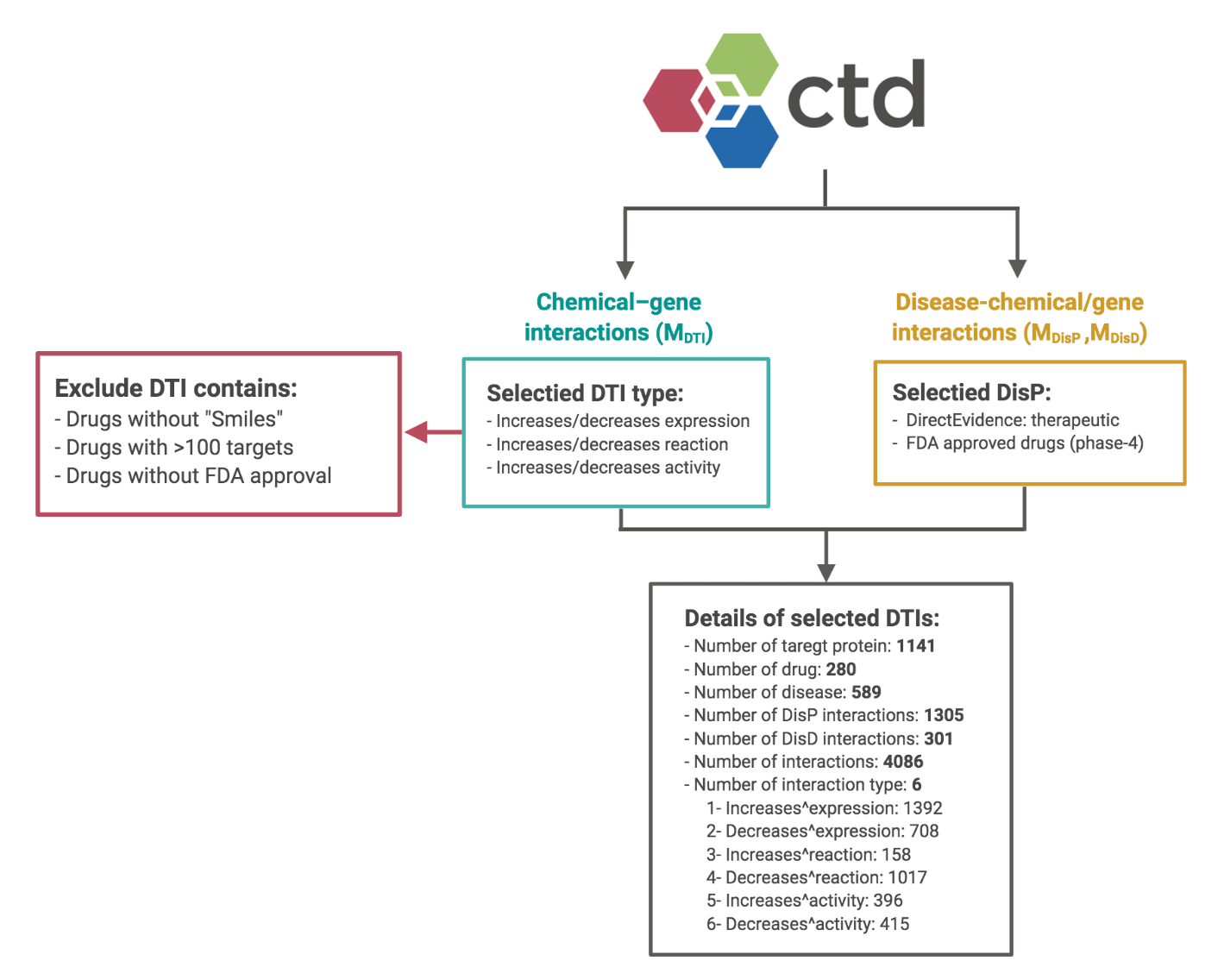


Supplementary Figure S1: steps performed to collect the dataset.

Supplement: Supplementary file 1 — Additional file 1. Fig. S1: steps performed to collect the dataset. [file 12859_2023_5317_MOESM1_ESM.docx]
